# Enhancement of TEX264-Mediated ER-Phagy Contributes to the Therapeutic Effect of Glycycoumarin against APA Hepatotoxicity in Mice

**DOI:** 10.3390/biomedicines9080939

**Published:** 2021-08-02

**Authors:** Mingzhu Yan, Zhi Wang, Tianji Xia, Suwei Jin, Yongguang Liu, Hongbo Hu, Qi Chang

**Affiliations:** 1 Institute of Medicinal Plant Development, Chinese Academy of Medical Sciences, Peking Union Medical College, Beijing 100193, China; mzyan@implad.ac.cn (M.Y.); wangzhimax@163.com (Z.W.); m13521515301@163.com (T.X.); jinsuwei0202@163.com (S.J.); liu17865562096@163.com (Y.L.); 2 Beijing Advanced Innovation Center for Food Nutrition and Human Health, College of Food Science and Nutritional Engineering, China Agricultural University, Beijing 100091, China

**Keywords:** acetaminophen, hepatotoxicity, ER-phagy, TEX264, ER stress, glycycoumarin

## Abstract

Acetaminophen (APA)-induced hepatotoxicity is coupled with the activation of autophagy. We sought to determine whether selective autophagy of the endoplasmic reticulum (ER), termed ER-phagy, is involved in APA hepatotoxicity and to explore its potential as a therapeutic target for APA-induced liver injury (AILI). APA (300 or 600 mg/kg) was administered to male C57BL/6N mice, with and without rapamycin, glycycoumarin (GCM) and *N*-acetylcysteine (NAC). The results demonstrated that ER-phagy accompanied with ER stress was activated after APA overdose. The dynamic changes of LC3 and TEX264 revealed that ER-phagy was induced as early as 6 h and peaked at 24 h following the APA injection. A delayed treatment with GCM, but not rapamycin, considerably attenuated a liver injury and, consequently, reduced its mortality. This is probably due to the inhibition of ER stress and the acceleration of liver regeneration via enhanced ER-phagy. Unlike the impaired hepatocyte proliferation and more severe liver injury in mice that received prolonged treatment with NAC, liver recovery is facilitated by repeated treatment with GCM. These findings suggest that TEX264-mediated ER-phagy is a compensatory mechanism against ER stress provoked by an APA overdose. A delayed and prolonged treatment with GCM enhances ER-phagy, thus serving as a potential therapeutic approach for patients presenting at the late stage of AILI.

## 1. Introduction

Acetaminophen (APA) is among the most commonly used over-the-counter antipyretic/analgesic medications. It is safe when taken at a therapeutic dosage. However, when abused, it can result in severe liver damage [1]. Since the first hepatotoxicity case that was reported in the 1960s [2], APA-induced acute liver injury (AILI) has emerged as the most frequent cause of acute liver failure in developed countries [3]. Previous pharmacokinetic studies identified phase II conjugating enzymes, particularly UDP-glucuronosyltransferase (UGT) and sulfotransferase (SULT), as the major metabolic enzymes involved in the conversion of APA to nontoxic compounds. When UGT and SULT become saturated following an APA overdose, the excessive APA is funneled into the cytochrome P450-dependent metabolic pathway (mainly CYP 2E1, CYP 3A4 and CYP 1A2), resulting in the formation of a highly reactive intermediate metabolite known as *N*-acetyl-para-benzo-quinone imine (NAPQI). Massive NAPQI depletes glutathione (GSH) in the mitochondria and subsequently covalently binds to proteins to form protein adducts. This, in turn, leads to mitochondrial oxidative stress and dysfunction, adenosine triphosphate (ATP) depletion, lipid peroxidation, DNA damage and, ultimately, hepatocyte necrosis [4]. *N*-Acetyl-L-cysteine (NAC) is the only approved antidote by the FDA for treating APA poisoning due to its ability in replenishing GSH, but NAC only exerts a therapeutic effect in the few hours after APA overdose [5]. Thus, developing a new therapeutic strategy, especially for patients presenting at the late phase of AILI, is critically needed. 

As a cellular adaptive mechanism in response to APA-induced hepatocyte damage, mitophagy is activated in APA hepatotoxicity to remove the damaged mitochondria and APA protein adducts [6,7,8]. APA-induced liver injury is further exacerbated by chloroquine but almost abolished in rapamycin-treated mice when observed at 6 h after chloroquine or rapamycin treatment. However, the type of autophagy and its potential role at a later stage of AILI is not confirmed. Several studies have reported that endoplasmic reticulum (ER) stress was involved in APA-mediated hepatotoxicity ta [9,10]. However, Hur et al. did not observe any evidence of unfolded protein response (UPR) activation after APA overdose in their study [11]. The data about the induction of ER stress by APA overdose have been inconclusive, probably because different doses of APA or sub-strains of mice were used in these studies. Nonetheless, these studies formed a strong basis for the hypothesis that ER-phagy is activated in APA hepatotoxicity, as recently demonstrated when damaged ER were selectively recognized and degraded by autophagy [12]. In mammals, several ER-phagy receptors have been identified: FAM134B, the long isoform of RTN3 (RTN3L), CCPG1, SEC62, ATL3 and TEX264—among which, TEX264 contributes the most to ER-phagy [13]. However, whether TEX264 is involved in APA hepatotoxicity and how TEX264-mediated ER-phagy contributes to AILI remain largely unknown.

Our previous studies have demonstrated that glycycoumarin (GCM), a natural coumarin compound isolated from licorice, was highly protective against alcoholic and nonalcoholic fatty liver disease [14,15] and APA-induced liver injury [16] in mice. Further investigation revealed that GCM eliminated APA hepatotoxicity by activating autophagy and, therefore, relieved oxidative stress. However, alleviating oxidative stress would be of limited benefit for patients in which liver injury has already been evident. Here, we show that TEX264-mediated ER-phagy is activated in APA hepatotoxicity, and a delayed treatment with GCM (after the oxidative stress has developed and NAC is no longer effective) inhibits ER stress and, subsequently, ameliorate APA-induced liver injury via enhancing ER-phagy. Furthermore, GCM also accelerates the compensatory liver regeneration process. This suggests the possibility of using an ER-phagy inducer as a therapeutic approach for AILI patients presenting at the late phase.

## 2. Materials and Methods

### 2.1. Chemicals and Reagents

APA was purchased from Cayman Chemical (Ann Arbor, MI, USA). Glycycoumarin (GCM, purity > 99%) was obtained from BioBioPha (Kunming, Yunnan, China). NAC and chloroquine diphosphate salt (CQ) were obtained from Sigma-Aldrich (St. Louis, MO, USA). Rapamycin (RA) was purchased from MedChem Express (Monmouth Junction, NJ, USA).

### 2.2. Animals and Treatments

Six-week-old male C57BL/6N mice (18–20 g) were purchased from the Charles River Laboratories (Beijing, China). Nrf2 knockout (KO) mice of C57BL/6J background and their wild-type littermates were kindly provided by Professor Siwang Yu, Peking University School of Pharmaceutical Sciences (Beijing, China). They were housed in groups of five to six per cage in plastic cages that were furnished with corncob bedding. Additionally, they were kept under controlled conditions of temperature (23 ± 2 °C), humidity (60% ± 5%) and 12-h light/dark cycles (lights on at 08:30 a.m.). The standard laboratory chow and water were made available ad libitum. All the animals were allowed to acclimatize for one week before the experiments were carried out.

**Figure 1:**
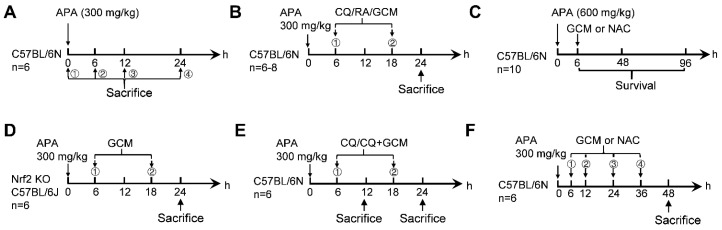
Experimental design. Mice were fasted overnight for 16 h before intraperitoneally (i.p.) injected with APA. Blood and liver tissues were collected at the time point of sacrifice. (**A**) The time–course experiment. (**B**) Autophagic flux confirmation experiment. (**C**) The survival experiment. (**D**) The experiment for the investigation of Nrf2. (**E**) The verification experiment. (**F**) The long-term treatment experiment. APA, acetaminophen; CQ, chloroquine diphosphate salt; RA, rapamycin; GCM, glycycoumarin; NAC, *N*-Acetyl-L-cysteine; KO, knockout.

The mice were divided into groups randomly (*n* = 6–10/group). They were made to fast overnight for 16 h before intraperitoneally (i.p.) injected with APA (prepared in 55 °C warm phosphate-buffered saline). The experimental design for all the treatment is shown in Figure 1. At the time point indicated in each experiment, the animals were weighed and then anesthetized with pentobarbital sodium (80 mg/kg, i.p.). Blood samples were collected from the abdominal aorta, and the animal were euthanized by cervical dislocation. The livers were quickly removed, washed with saline and weighed.

(i) In the time–course experiment, four groups of C57BL/6N mice (*n* = 6/group) were used. Their blood and liver tissues were collected at 0, 6, 12 and 24 h after the APA (300 mg/kg) injection, respectively (Figure 1A).
(ii) For the autophagic flux confirmation experiment, eight groups of C57BL/6N mice were used (*n* = 6–8/group): the phosphate-buffered saline (PBS) group; the APA, CQ, RA and GCM alone groups and APA combined with CQ and RA and GCM groups. CQ (60 mg/kg), RA (2 mg/kg) or GCM (100 mg/kg; dissolved in Tween-80:saline (2:98, *v*/*v*)) were administered by i.p. injection 6 and 18 h after APA overdose to inhibit or activate autophagy (Figure 1B). The blood and liver tissues were collected 24 h after APA overdose.
(iii) As for the survival experiment, four groups of C57BL/6N mice were used (*n* = 10/group): APA alone group, APA treated with GCM (100 mg/kg) and NAC (100 and 300 mg/kg) groups. GCM and NAC were injected in mice 6 h following a lethal dose of APA (600 mg/kg) (Figure 1C). The survival of mice was observed until 96 h.
(iv) To study the role of Nrf2 in the protective effect of GCM, Nrf2 KO mice of C57BL/6J background and their wild-type littermates were used (*n* = 6/group) as follows: the PBS group, APA-treated group, in which mice were injected with 300 mg/kg APA, and APA + GCM group, in which APA-treated mice were injected with GCM (100 mg/kg) 6 and 18 h after the APA overdose.
(v) In the verification experiment, five groups of C57BL/6N mice (*n* = 6/group) were used as follows: the PBS group, 12- and 24-h APA + CQ groups and 12- and 24-h APA + CQ + GCM groups. Mice were injected with CQ (60 mg/kg) or CQ + GCM (100 mg/kg) 6 and 18 h after APA administration. Their blood and liver tissues were collected 12 and 24 h after APA overdose.
(vi) In the long-term treatment experiment, four groups of C57BL/6N mice were used as follows: the PBS group, APA alone group, APA treated with GCM and NAC group. GCM (100 mg/kg) or NAC (100 mg/kg) were administered repeatedly at 6, 12, 24 and 36 h after APA (300 mg/kg) overdose (Figure 1F) The blood and liver tissues were collected 48 h following the APA challenge.

### 2.3. Measurement of Alanine Aminotransferase and Aspartate Aminotransferase

Whole blood samples were centrifuged at 1000× *g* for 15 min to yield a serum. Serum alanine aminotransferase (ALT) and aspartate aminotransferase (AST) activities were measured by an automatic biochemical analyzer (Beckman Coulter AU480, Brea, CA, USA) using commercially available reagent kits from Biosino Bio (Beijing, China).

### 2.4. Histology, Immunohistochemistry and Immunofluorescence

Liver tissues were fixed in 4% paraformaldehyde-buffered solution for 24 h, embedded with paraffin and then after cut into 5-μm-thick sections. To observe the pathological changes in the liver tissues, some tissue sections were stained with hematoxylin and eosin (H&E). APA-induced hepatic necrosis was quantified by ImageJ 1.46r software (NIH, Bethesda, MD, USA) and expressed as a percent of necrosis relative to the examined total tissue area. As for PCNA immunohistochemistry, the liver sections were stained with a rabbit monoclonal anti-PCNA antibody at a 1:4000 dilution ratio. For quantification of PCNA staining, images of the stained liver sections were captured. Then, PCNA-positive hepatocyte nuclei were counted in 6 representative 200× sections and expressed as the total number of PCNA-positive cells. For immunofluorescence analyses, the paraffin-embedded sections were stained with a rabbit anti-mouse LC3 antibody at a dilution of 1:200. The sections were counterstained with 4′,6-diamidino-2-phenylindole dihydrochloride (DAPI) before being mounted.

### 2.5. Western Blot Analysis

Liver tissues were homogenized and lysed in RIPA buffer (Solarbio, Beijing, China), containing 1% phosphatase and a protease inhibitor cocktail (CWBIO, Beijing, China). Thirty to fifty micrograms of the denatured proteins and 3 μL of multicolor protein marker (CWBIO, Beijing, China) were subjected to SDS-PAGE and electrotransferred onto a nitrocellulose membrane (0.2 μm). After incubation in 5% nonfat milk, the membranes were washed 3 times and incubated with the specific primary antibodies overnight at 4 °C. Primary antibodies specific for CHOP (Cat# 2895S), eIf2α (Cat# 5324S), p-eIf2α (Cat# 3398S), ATF-6 (Cat# 65880), IRE1α (Cat# 3294), CyclinD1 (Cat# 55506) and proliferating cell nuclear antigen (PCNA, Cat# 13110) were purchased from Cell Signaling Technology (Denver, MA, USA) and were used at a dilution of 1:1000. The primary antibodies specific for p-IRE1 (Cat# AP0878), XBP1 (Cat# A1731), LC3 (Cat# A19665) and β-actin (Cat# AC026) were purchased from ABclonal (Wuhan, Hubei, China) and were used at a 1:1000 dilution except for β-actin, which was diluted at 1:5000. The primary antibodies specific for Beclin1 (Cat# 11306-1-AP), *SQSTM1*/*p62* (Cat# 18420-1-AP) and TEX264 (Cat# 25858-1-AP) were obtained from Proteintech (Wuhan, Hubei, China) and were used at 1:1000 dilution. Wash three times for 5 min each. Later on, they were incubated with the appropriate secondary antibodies for 1 h at room temperature. Horseradish peroxidase-conjugated goat anti-rabbit immunoglobulin G (Cat# 485) and anti-mouse immunoglobulin G (Cat# 330) secondary antibodies were purchased from MBL International Corporation (Woburn, MA, USA) and were diluted at 1:5000 in blocking solution. Protein expression was visualized using an enhanced chemiluminescence method in a gel imaging system (Bio-Rad, Hercules, CA, USA). The signal intensities were normalized to the corresponding total protein or β-actin.

### 2.6. Quantitative Real-Time PCR

Total RNA was isolated from liver tissue using the TRlzol reagent (Cwbiotech, Beijing, China) according to the manufacturer’s instructions. A total of 5000 ng of RNA was used to perform reverse transcription with the *TransScript*^®^ kit (TransGen, Beijing, China). The cDNA was subjected to quantitative real-time PCR analysis on a Roche LightCycler 96 system using the *TransStart*^®^ Top Green SuperMix (TransGen, Beijing, China). The housekeeping gene *GAPDH* (Sangon Biotech, B661304, Shanghai, China) was used as an internal control. The primers used in real-time PCR are shown in Table 1.

### 2.7. Transmission Electron Microscopy

Before euthanasia, a small portion of liver tissue in anesthetized mice was fixed in 2.5% glutaraldehyde for 0.5 h. The samples were further cut into 1 × 1-mm^2^ slices and fixed using the same fixative for 2 h. After the post-fixation in 1% osmium tetroxide and 1% tannic acid, and the samples were embedded with epoxy resin and cut into 70-nm ultrathin sections. The sections were double stained with uranyl acetate and the lead citrate before being observed under an electron microscope (HT7700, Tokyo, Japan) at 80 kV.

### 2.8. Statistical Analysis

The results are presented as the mean ± standard errors of mean (SEM). The quantification of Western blot bands was performed using ImageJ software. Statistical comparisons were performed by Student’s *t*-test, one-way or two-way ANOVA, followed by Tukey’s post hoc test using SPSS19.0 (IBM Corp., Armonk, NY, USA). *p* < 0.05 was considered statistically significant. Graphs were drawn using GraphPad Prism (version 8.0 for Windows).

## 3. Results

### 3.1. ER-Phagy Is Activated and Correlates with ER Stress in APA Hepatotoxicity

**Figure 2:**
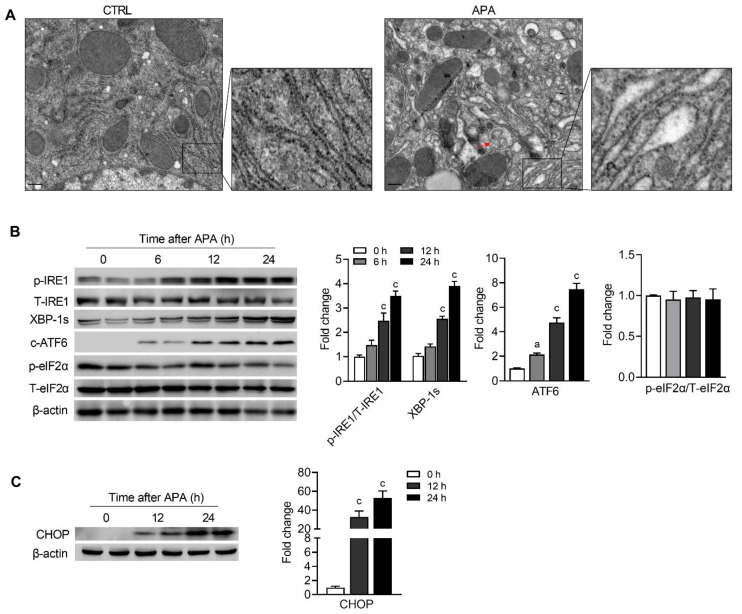
ER-phagy is activated and is accompanied with an unfolded protein response in APA hepatotoxicity. Mice were fasted for 16 h before injection with PBS or APA (300 mg/kg). At 6, 12 and 24 h post-APA, liver tissues were collected. (**A**) Representative TEM images of hepatocytes in mice liver at 24 h following PBS or APA injection. Scale bar = 400 nm. Arrows denote ER-selective autophagosomes. Immunoblot analysis for (**B**) p-IRE1, total IRE1, the sliced XBP-1, the cleavage of ATF6, p-eIF2α, total eIF2α and (**C**) CHOP at the indicated time points. Quantification of the phosphorylated proteins normalized to the corresponding total proteins bands, with the other protein bands normalized to the respective β-actin bands and, again, to the mean value of the control (CTRL) group are shown on the right. Data are presented as mean ± SEM. a: *p* < 0.05 and c: *p* < 0.001 compared with the vehicle control.

Overnight fasted male C57BL/6N mice were injected i.p. with a toxic dose of APA (300 mg/kg) to induce APA hepatotoxicity. Three hours after injection, mice were fed ad libitum until the time of sacrifice. Starvation was previously shown to induce activation of the ER-phagy in mice livers [17]. A vehicle control was therefore used to ensure that, if ER-phagy was activated, it was a direct result of APA overdose. To observe the ER morphology intuitively, liver samples were processed for transmission electron microscopy (TEM) at 24 h after APA overdose. In the hepatocytes of control mice, normally, narrow ER closely surrounded the mitochondria and nucleus and additionally built a network of ER membranes that stretched throughout the cytoplasm (Figure 2A, left panel). Numerous ribosomes were attached to the outer membranes of the ER. Unlike the relatively normal ER observed in the controls, APA induced a deformed ER network with fragmented and swollen ER structures (Figure 2A, right panel). The number of ribosomes in the rough ER appeared to decrease after APA treatment. These morphological changes indicated that ER was damaged by APA overdose. In addition, we observed autophagosomes that enclosed ER membranes in the damaged hepatocytes (Figure 2A, red arrow), implying that impaired ER might induce ER-phagy in APA-induced liver injuries. We next assessed whether ER stress was accompanied by the activation of ER-phagy during the progression of AILI in mice, as recently demonstrated for the role of ER stress in triggering autophagy [18]. Under ER stress conditions, the unfolded protein response (UPR) is transduced by three major sensors (IRE1, PERK and ATF6) to handle unfolded proteins [19]. The phosphorylation of IRE1 and ATF6 cleavage were observed as early as 6 h following the APA overdose (Figure 2B). As a downstream target of IRE1, the levels of spliced XBP1 increased after drug treatment (Figure 2B). At 12 and 24 h, higher levels of phosphorylated IRE1, spliced XBP1 and cleaved ATF6 were detected by immunoblotting analyses (Figure 2B). However, we did not observe the activation of the PERK-eIF2α arm, as indicated by the relatively unchanged levels of phosphorylated eIF2α (Figure 2B). CHOP, whose protein expression was 32- and 53-fold elevated 12 and 24 h after APA administration, respectively (Figure 2C), acted as a joint point of the IRE1 and ATF6 signaling pathways. Combined, these results suggested that ER-phagy induced by APA overdose was accompanied by ER stress.

### 3.2. APA Overdose Induces ER-Phagy Involving TEX264

**Figure 3:**
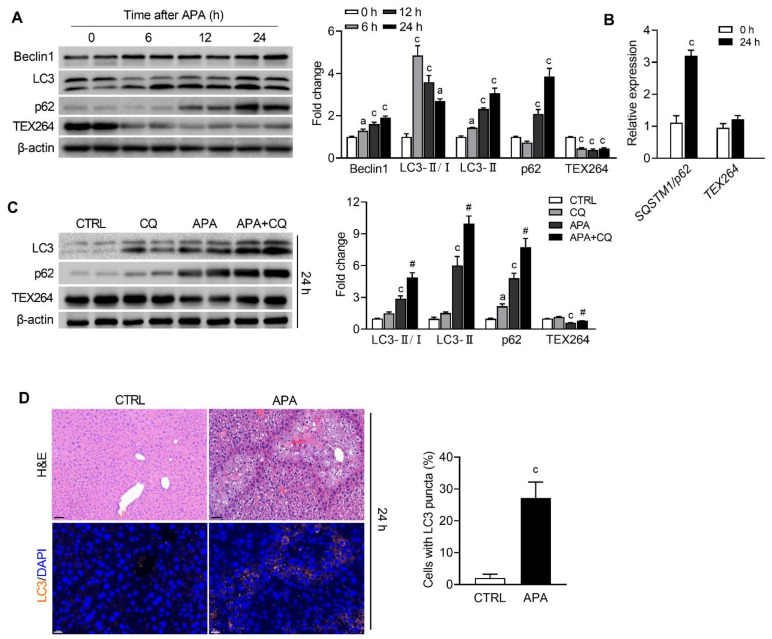
An APA overdose induces TEX264-mediated ER-phagy. Mice were injected with PBS or APA (300 mg/kg), and liver tissues were harvested for analysis at 6, 12 and 24 h after APA overdose. (**A**) Immunoblot analysis for Beclin1, LC3, p62, TEX264, and the corresponding densitometry normalized to β-actin. (**B**) Real-time RT-PCR was performed on mice treated with PBS or APA for 24 h. The mRNA expression of *SQSTM1*/*p62* and *TEX264* (6–8 mice per group). (**C**) Mice were injected with CQ (60 mg/kg) 6 and 18 h after APA, and the liver tissues were collected at 24 h post-APA. Immunoblot analysis for LC3, p62, TEX264 and the corresponding densitometry normalized to β-actin. (**D**) Representative images of hematoxylin–eosin (H&E) staining (upper panel, scale bar = 50 μm), and immunofluorescence staining for LC3 (orange; co-stained with DAPI, blue) (lower panel, scale bar = 20 μm) at 24 h following the APA overdose. Data are presented as the mean ± SEM. a: *p* < 0.05 and c: *p* < 0.001 compared with the vehicle control. ^#^ *p* < 0.05 compared with the corresponding APA-treated group.

We next determined the kinetics of the TEX264 expression, as TEX264 is identified as a major ER-phagy receptor [13]. In addition, the time–course of the autophagy response was monitored to exclude the interference of starvation. Beclin1 and LC3-II were markedly increased in APA-treated mice compared to the controls at 6, 12 and 24 h after APA (Figure 3A), implying activated autophagy. A decreased p62 protein expression further confirmed that the autophagic flux was accelerated at 6 h (Figure 3A). Curiously, the expression of p62 was significantly upregulated 12 and 24 h following the APA overdose (Figure 3A). To determine whether the degradation of p62 was blocked or the biosynthesis was increased, we measured the mRNA expression of *SQSTM1*/*p62* by real-time PCR. The *SQSTM1/p62* gene was robustly induced following the APA treatment (Figure 3B), indicating the transcriptional activation of *SQSTM1*/*p62*. Unlike p62, TEX264, a receptor for autophagic degradation of the ER, was reduced in a time-dependent manner (Figure 3A), while the mRNA levels of *TEX264* slightly increased in APA-treated mice compared to the controls (Figure 3B). Combined, the data suggested that autophagy, especially TEX264-mediated ER-phagy, was activated after the APA overdose. To verify this, we blocked the autophagic flux with CQ. The expression of LC3-II, p62 and TEX264 increased after the CQ treatment compared with the control (Figure 3C), and all of them accumulated further in CQ- and APA-treated mice compared with those treated with APA alone (Figure 3C). This result signifies that ER-phagy flux is normal in AILI. To examine the anatomical location of ER-phagy activation, we analyzed LC3 by immunofluorescence. A punctuated pattern of LC3 fluorescence was observed in the hepatocytes localized in zone 3 of the liver lobules (Figure 3D), the area accompanied by APA-induced liver damage.

### 3.3. GCM, but Not Rapamycin, Enhances ER-Phagy and Alleviates APA Hepatotoxicity

**Figure 4:**
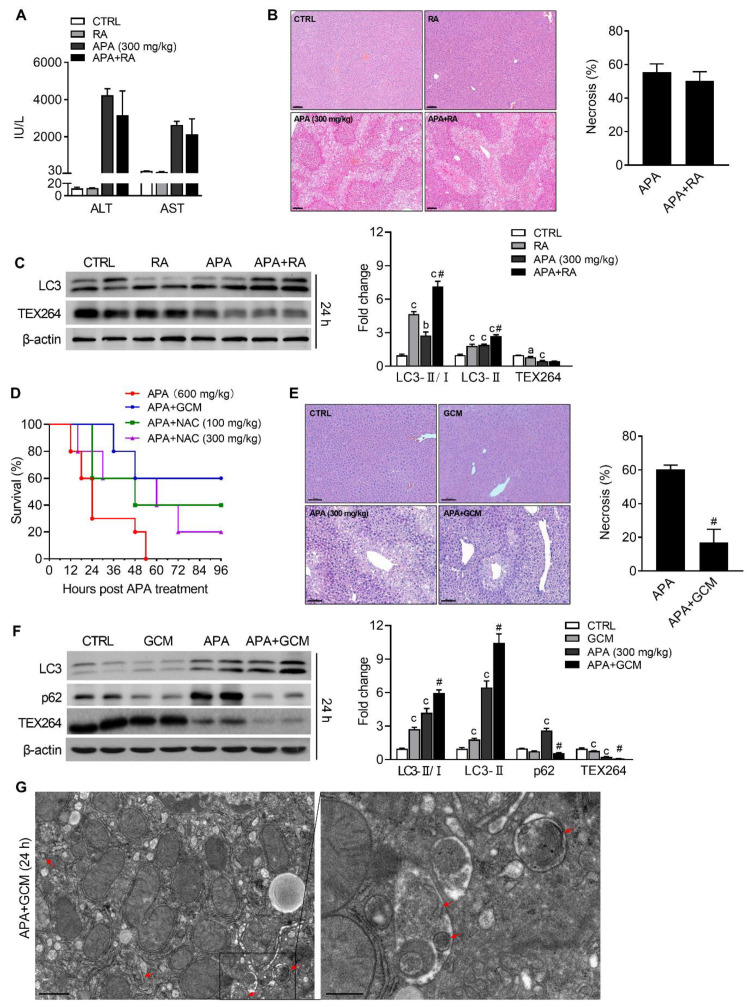
ER-phagy enhanced by GCM protects against APA-induced liver injuries. (**A**–**C**), Mice were treated with APA (300 mg/kg) followed by rapamycin (RA; 2 mg/kg) 6 and 18 h after APA. Blood and livers were harvested for analysis at 24 h. (**A**) Serum ALT and AST levels. (**B**) Representative H&E staining of liver tissues (left panel) and quantification of the centrilobular necrotic areas (right panel). (**C**) Immunoblot analysis for LC3, TEX264 (left panel) and the corresponding densitometry normalized to β-actin (right panel). (**D**) Mice were injected i.p. with GCM or NAC 6 h after a lethal dose of APA (600 mg/kg). Survival was evaluated for 96 h post-APA. (**E**–**G**) Mice were treated GCM (100 mg/kg) 6 and 18 h after APA (300 mg/kg) overdose. Livers were collected at 24 h. (**E**) Representative H&E staining of the liver tissues (left panel) and the quantification of centrilobular necrotic areas (right panel). Scale bar = 100 μm. (**F**) Immunoblot analysis for LC3, p62 and TEX264 (left panel) and the corresponding densitometry normalized to β-actin (right panel). (**G**) Representative TEM images of hepatocytes in mice livers at 24 h following the APA and GCM treatments. Scale bar = 400 nm. Arrows denote ER-selective autophagosomes. Data are presented as the mean ± SEM. a: *p* < 0.05, b: *p* < 0.01 and c: *p* < 0.001 compared with the vehicle control. ^#^ *p* < 0.05 compared with the corresponding APA-treated group.

In order to assess the role of ER-phagy in APA-induced liver injuries, we utilized autophagy-inducer rapamycin. As mitochondrial oxidative stress and the corresponding mitophagy develop early in AILI, the mice were therefore injected with rapamycin 6 and 18 h after the APA challenge to enhance the ER-phagy. However, no obvious protection was seen after the rapamycin treatment, as revealed by the slightly decreased ALT and AST levels (Figure 4A) and necrotic areas (Figure 4B). Though immunoblotting analyses showed that the ratio of LC3-II/LC3-I was elevated in rapamycin-treated mice (Figure 4C), the expression of TEX264 remained unchanged in mice treated with and without rapamycin after an APA overdose (Figure 4C), supporting the results above. We next used another activator of autophagy, GCM, which was tested in APA hepatotoxicity in our previous study [16]. The administration of GCM (100 mg/kg) at 6 h post the lethal dose of APA (600 mg/kg) significantly reduced the mortality, with a survival rate of 60% (Figure 4D). Meanwhile, NAC at the same dose only improved the survival rate to 40%, and a higher dose of NAC (300 mg/kg) showed an even worse survival rate of 20% (Figure 4D), suggesting that the protective effect of GCM at the late phase of AILI was not due to the inhibition of mitochondrial oxidative stress. The hepatic necrotic area remarkably decreased from 60% in the APA group to 17% by the GCM treatment at 6 and 18 h after the toxic dose of APAP (300 mg/kg), as revealed by H&E staining (Figure 4E). Similar to that in rapamycin-treated mice, the ratio of LC3-II/LC3-I obviously increased after GCM intervention (Figure 4F). However, unlike the rapamycin treatment, the GCM administration reduced the expression of p62 and TEX264 further compared with the corresponding control or APA-treated mice (Figure 4F). In addition, autophagosomes that enclosed the ER membranes in the hepatocytes were more than those observed in APA-treated mice (Figure 4G vs. Figure 2A). Collectively, these results suggest that TEX264-mediated ER-phagy, which was enhanced by GCM in our study, might be protective in APA-induced liver injuries.

### 3.4. ER-Phagy Enhanced by GCM Suppresses ER Stress in APA Hepatotoxicity

**Figure 5:**
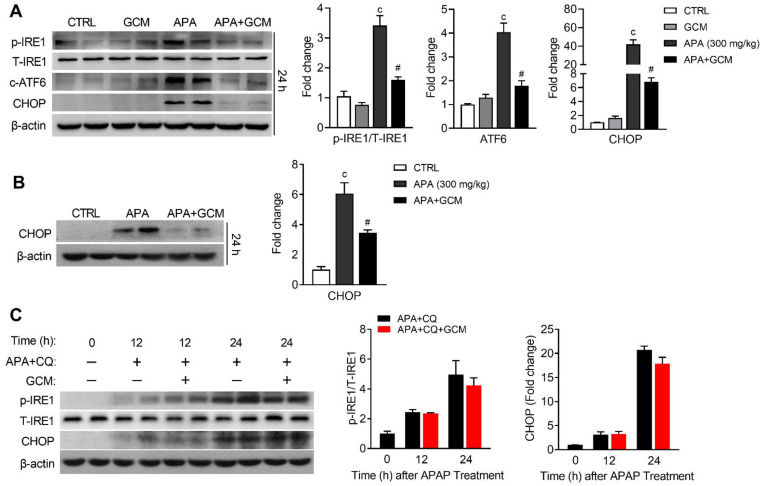
GCM suppresses ER stress via enhancing ER-phagy. (**A**,**B**) Mice were treated with 300 mg/kg of APA, followed by 100 mg/kg of GCM 6 and 18 h after APA. Livers were harvested at 24 h for analysis. (**A**) Immunoblot analysis for p-IRE1, toral IRE1, the cleavage of ATF6 and CHOP (left panel) and the corresponding densitometry normalized to the corresponding total protein or β-actin (right panel). (**B**) Immunoblot analysis for CHOP at 24 h after APA in Nrf2 knockout mice (left panel) and the corresponding densitometry normalized to β-actin (right panel). (**C**) Mice were treated with APA, followed by CQ and GCM treatment. Immunoblot analysis of a whole-liver homogenate for p-IRE1 CHOP at 12 and 24 h after the APA overdose (left panel) and the corresponding densitometry normalized to the corresponding total protein or β-actin (right panel). Data are presented as the mean ± SEM. c: *p* < 0.001 compared with the vehicle control. ^#^ *p* < 0.05 compared with the corresponding APA-treated group.

To understand the mechanism underlying the protective effect of GCM-induced ER-phagy in AILI, the IRE1 and ATF6 pathways were assessed by Western blotting. The GCM treatment significantly inhibited the phosphorylation of IRE1 and the cleavage of ATF6 (Figure 5A), which was triggered by APA overdose. As such, the expression of CHOP was reduced in GCM-treated mice (Figure 5A). Our previous studies demonstrated that GCM ameliorated alcohol-induced hepatotoxicity via the activation of Nrf2 [15]. Thus, we investigated the expression of CHOP in Nrf2 KO mice, in which GCM could not alleviate the oxidative stress and ER stress induced by APA by activating Nrf2. As expected, the GCM treatment could still inhibit the CHOP elevation induced by an APA overdose in Nrf2 KO mice (Figure 5B). To further investigate whether it was the induction of ER-phagy or GCM itself that was responsible for the ER stress suppression, we used CQ to suppress ER-phagy. Our results showed that the inhibition of p-IRE1 and CHOP caused by GCM was abrogated in the autophagy-retarded mice (Figure 5C). This confirmed that ER-phagy enhanced by GCM inhibits ER stress in AILI.

### 3.5. GCM Accelerates Liver Regeneration after APA Overdose

**Figure 6:**
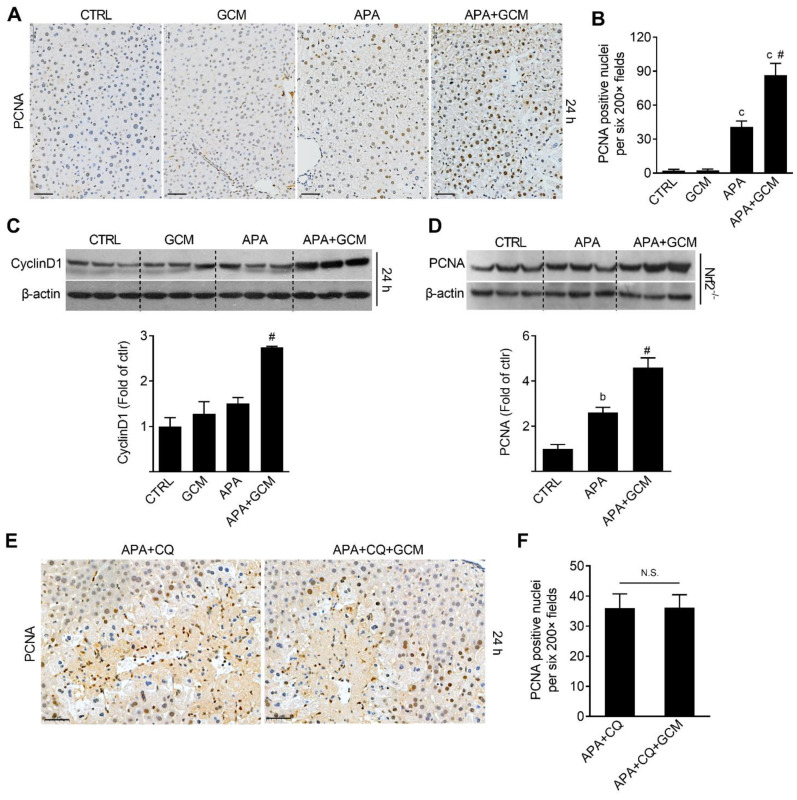
Glycycoumarin (GCM) promotes liver regeneration in wild-type and Nfr2 knockout mice. Mice were treated with 300 mg/kg of APA followed by 100 mg/kg of GCM 6 and 18 h after APA, and the livers were harvested for analysis 24 h after the APA overdose. (**A**) Representative immunohistochemical staining images of PCNA in liver tissues. (**B**) Quantification of the PCNA-positive hepatocyte nuclei. Immunoblot analysis of the whole-liver homogenate for (**C**) CyclinD1 in wild-type mice and (**D**) PCNA in Nrf2 knockout mice and the corresponding densitometry normalized to β-actin (**E**,**F**). Mice were treated with 300 mg/kg of APA followed by a CQ or/and GCM treatment 6 and 18 h after APA. (**E**) Representative immunohistochemical staining images of PCNA in liver tissues. (**F**) The quantification of PCNA-positive hepatocyte nuclei. Scale bar = 50 μm. Data are presented as the mean ± SEM. b: *p* < 0.01; c: *p* < 0.001 compared with the vehicle control. ^#^ *p* < 0.05 compared with the corresponding APA-treated group. N.S.: No significance.

Since the kinetics of autophagy induction showed that the amount of TEX264 was reduced in a time-dependent manner, from 6 h to 24 h following the APA challenge, we hypothesized that late-phase liver regeneration might be associated with ER-phagy. In order to test this, PCNA and CyclinD1, which are established markers of hepatocyte proliferation, were assessed in mice treated with or without GCM following the APA challenge. As expected, PCNA-positive hepatocyte nucleus (Figure 6A,B) and the expression of CyclinD1 increased in the APA group 24 h after the APA overdose relative to the vehicle control (Figure 6C). The GCM intervention elevated the two makers further. The quantitative analysis showed that the number of PCNA-positive hepatocytes and the levels of CyclinD1 increased by 72% and 82%, respectively (Figure 6B,C). Our previous studies demonstrated the potential of GCM in treating alcohol-induced hepatotoxicity via the activation of Nrf2. However, the data revealed that GCM could still elevate the PCNA level when compared with APA treated alone in Nrf2 KO mice (Figure 6D), ruling out the role of Nrf2 in protecting mice from AILI. In addition, the CQ treatment abolished the accelerated liver regeneration caused by GCM (Figure 6E,F), suggesting the involvement of autophagy in this process.

**Figure 7:**
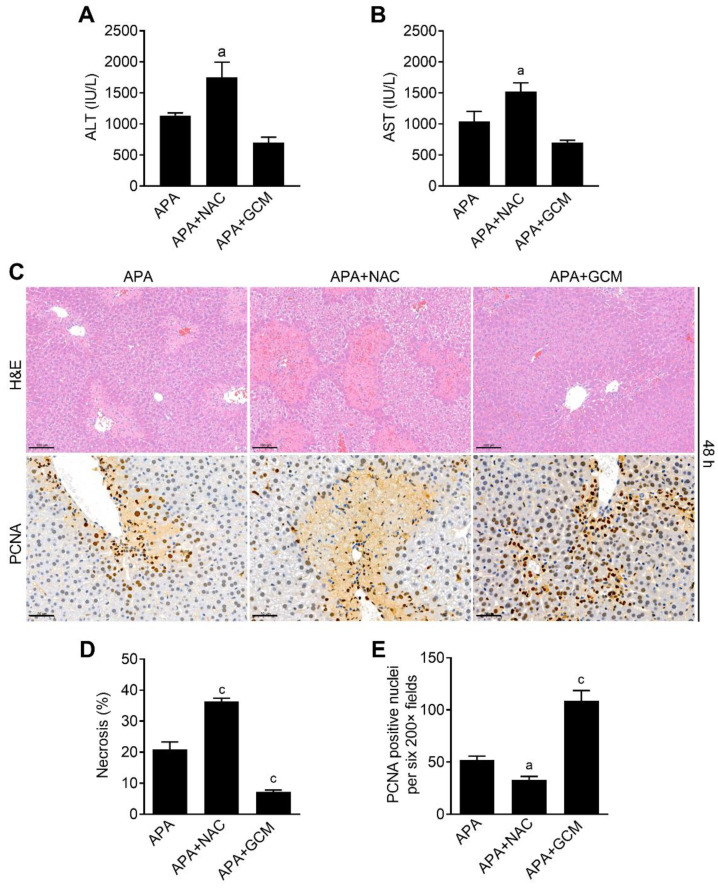
Prolonged treatment with glycycoumarin (GCM) facilitates liver recovery from APA hepatotoxicity. Animals were treated with 300 mg/kg of APA followed by 100 mg/kg of GCM or 100 mg/kg of NAC at 6, 12, 24 and 36 h after APA, and the blood and livers were harvested for analysis 48 h after the APA overdose. (**A**) The serum ALT and (**B**) AST levels and (**C**) histological characterization (upper panel, scale bar = 100 μm) and IHC staining for PCNA (lower panel, scale bar = 50 μm) in liver sections at 72 h were evaluated. (**D**) The percentage of hepatic necrosis areas and (**E**) PCNA-positive hepatocytes per photomicrographs were also quantified. Quantitative data are presented as the mean ± SEM. a: *p* < 0.05, c: *p* < 0.001 compared with the APA group.

Since a prolonged treatment with NAC is toxic and impairs liver regeneration [20], we then asked whether GCM is superior to NAC when it was administered as a long-term treatment at the late stage of AILI. To explore this, the mice were repeatedly treated with NAC (100 mg/kg) or GCM (100 mg/kg) at 6, 12, 24 and 36 h after the APA overdose. In accordance with the previous studies, forty-eight hours after the APA challenge, the mice in the NAC therapy group showed significantly higher serum ALT and AST levels and a larger hepatic necrosis area in comparison to the APA-treated alone (Figure 7A–D). This might be a result from the impaired resolution of hepatic damage, as the number of PCNA-positive hepatocytes was less than that in the APA group (Figure 7C,E). On the contrary, a prolonged treatment with GCM alleviated the APA hepatotoxicity, as demonstrated by the declined ALT and AST levels (Figure 7A,B). Additionally, the histological analysis indicated reduced necrotic areas (Figure 7C,D) and an increased number of PCNA-positive hepatocytes (Figure 7C,E) at 48 h in the livers of the GCM-treated mice. Overall, GCM as an ER-phagy inducer accelerated liver regeneration and exhibited a therapeutic potential superior to that of NAC for the treatment of late-stage AILI.

## 4. Discussion

APA-induced hepatotoxicity is common in developed nations and is also increasingly reported in other less-developed countries [4]. NAC is the optimal choice to treat AILI. However, the resultant adverse effects and narrow therapeutic window limits its application. As such, it is necessary to be thorough in discovering the mechanisms of APA hepatotoxicity so that better treatments with fewer side effects can be developed. In the present study, we identified a previously unreported mechanism of ER-phagy that the liver coordinate ER stress induced by an APA overdose. Our results also suggest that treatment with GCM beyond the initiation of oxidative stress was protective against APA hepatotoxicity by enhancing TEX264-mediated ER-phagy.

Mitochondria are the main target of APA; hence, mitochondrial oxidative stress plays a vital role in the progression of APA hepatotoxicity. Mitochondrial quality control and the removal of damaged mitochondria through mitophagy have become a potential strategy to treat APA-induced liver injuries [21]. Though the injury-initiated treatment of rapamycin activates autophagy and protects mice from APA hepatotoxicity [6], we did not observe its therapeutic effect when administered at 6 and 18 h following an APA overdose (Figure 4A–C). This is because mitochondrial oxidative stress develops early after the APA overdose, so targeting mitochondrial may not be the optimal choice for patients presenting at a later phase of AILI. It has been previously shown that ER stress is activated and occurs late in APA-induced liver injuries, after GSH is depleted and after oxidative stress has already developed [9], suggesting that the means to inhibit ER stress may prevent the downstream death signaling pathways. However, this aforementioned result was achieved in mice treated with APA by gavage, not by the conventionally used intraperitoneal route. In the present study, we found that the IRE1-XBP1 and ATF6 arms, but not the PERK-eIF2α arm of the UPR, were activated as early as 6 h following an intraperitoneal injection with APA and peaked at 24 h (Figure 2B,C). These results are in agreement with previous studies that demonstrated the 4-phenylbutylic acid significantly ameliorated the massive hepatocyte apoptosis/necrosis in mice, accompanied with the decreased cleavage of ATF6 and hepatic Xbp1 mRNA splicing [22,23].

To maintain the cellular homeostasis, the ER stress-mediated autophagy and ER-phagy were induced by ER stress. The former one generates autophagosomes mainly to remove aggregated or misfolded proteins, while the ER-phagy is characterized by the formation of autophagosomes with ER-derived membranes [24]. The morphological observations by TEM showed that ER was severely damaged after the APA overdose (Figure 2A). Autophagosomes that encapsulated ER membranes were also observed in the present study. In addition to the core autophagy machinery, ER-phagy receptors are required in modulating ER-phagy. Recently, TEX264 was identified to be the most contributory receptor in mammalian ER-phagy [13]. Expectedly, TEX264 was degraded by ER-phagy 6 h after APA, along with the autophagy substrate p62. When the time–course was extended, the level of TEX264 continued to decline while p62 started to increase significantly. As the treatment with CQ confirmed that the autophagic flux was not blocked, this might be due to the increased synthesis of p62 (Figure 3B). The gene expression of *SQSTM1*/*p62* has been reported to be trans-regulated by several transcription factors [25]; among which, Nrf2, NF-kB and the transcription factor CHOP/DDIT3 are the likely regulators of *SQSTM1*/*p62*, as they were all activated in APA-induced liver injuries [26,27].

The start-up phase of ER-phagy, as measured by increased LC3 II and decreased TEX264, was activated as early as 6 h after the APA overdose and continued until 24 h. We therefore hypothesized that targeting ER-phagy might be protective at the late phase of AILI. In the present study, rapamycin, an mTOR inhibitor, induced an increase in LC3 mobility but had no impact on the TEX264 expression and the extent of the liver injury as well. In contrast, GCM administered at 6 and 18 h after APA reduced the APA-induced hepatotoxicity and mortality. Distinct from rapamycin, GCM enhanced the TEX264-mediated ER-phagy by which it inhibited ER stress. This may explain the differences in therapeutic effects between these two autophagy inducers. Considering that TEX264 dramatically decreased 24 h after the APA overdose, we reasoned that the mechanisms related to liver regeneration were also engaged. Testing for liver regeneration demonstrated that 24 h post-challenge, when hepatocyte proliferation was initiated in APA-treated mice, proliferation was more pronounced in GCM-treated mice. The abilities of GCM to inhibit ER stress and to promote liver regeneration in APA hepatotoxicity were also seen in Nrf2 knockout mice, ruling out the involvement of Nrf2 in ameliorating liver injuries, as previous reported [15]. Further investigation demonstrated that the increase in proliferated hepatocytes was due to the enhancement in autophagy.

Since NAC is the only recommended antidote by the FDA for APA overdose patients, we compared the therapeutic effect of GCM with that of NAC. The most recognized mechanism of protection by NAC is providing a synthetic precursor for the synthesis of GSH in hepatocytes, which could directly detoxify NAPQI [28]. This prevents excessive NAPQI generation and, hence, blocks the covalent modification of the cellular proteins. Consequently, NAC prevents the initiation of APA toxicity at the metabolism phase. Additionally, NAC has protective effects when given at the oxidative injury phase when hepatocellular GSH is depleted and reactive oxygen species and peroxynitrite are generated in mitochondria [29]. Though not as effective as at the metabolism phase, NAC administration at the APA injury phase could limit hepatocyte necrosis and, hence, improve the prognosis for APA overdose patients. Another speculated mechanism through which NAC may protect against APA-induced liver injury is inhibiting platelet accumulation and the subsequent platelet-mediated liver injuries. A recent study has reported that NAC reduces the size of von Willebrand factor (vWF) multimers and platelet-vWF string formation on endothelial cells [30]. The main problem associated with NAC is the decreased therapeutic effect when taken >8 h after the overdose [31,32]. When the early and most-treatable stage is missed, a liver transplantation is the only choice to improve patient survival [33]. Our study suggested that GCM is superior to NAC in treating APA-induced hepatotoxicity, particularly as a delayed treatment (Figure 4D). Some previous studies suggested that a prolonged treatment with NAC might be detrimental rather than protective in APA hepatotoxicity and could impair liver regeneration [20]. This is because ROS in the neutrophils activate macrophages, transforming into a reparative phenotype for liver repair [34]. The contradictory impact of NAC during the metabolic activation phase and the resolution phase was established in our study. Specifically, more pronounced liver damage and reduced hepatocyte proliferation were observed after NAC intervention at 6, 12, 24 and 36 h following the APA challenge. A prolonged treatment with GCM was still beneficial, probably due to the accelerated liver regeneration [35,36,37,38,39,40,41,42]. The mechanism through which GCM facilitated liver regeneration after GCM treatment was indistinct; the assumed mechanisms include an energy supplementation due to ER-phagy leading to recycling damaged and unwanted organelles. However, the precise role of ER-phagy in the resolution phase of APA-induced liver injury was not investigated in our study. Hence, further investigation is needed to address this issue, as liver regeneration is a critical determinant for the final outcome in patients of AILI.

## 5. Conclusions

In summary, TEX264-mediated ER-phagy is activated in APA-induced liver injuries and is associated with ER stress provoked by an APA overdose. GCM inhibits ER stress and promotes liver regeneration by enhancing TEX264-mediated ER-phagy in APA-treated mice. Additionally, our findings imply delayed and prolonged treatment with GCM as a potential therapeutic approach superior to NAC to treat patients at the late stage of AILI.

## Figures and Tables

**Table 1:** Primer sequences for real-time PCR.

Genes (Mouse)	Forward Primer (5′–3′)	Reverse Primer (5′–3′)
*TEX264*	GAGCTGATACAAGTGATGCAAG	GATTCACTGTAGCTGTGCTCG
*SQSTM1*/*p62*	GAACACAGCAAGCTCATCTTTC	AAAGTGTCCATGTTTCAGCTTC
*GAPDH*	GGTTGTCTCCTGCGACTTCA	TGGTCCAGGGTTTCTTACTCC

## Data Availability

The data presented in this study are available upon request from the corresponding author.

## Abbreviations

ALT	Alanine aminotransferase
AILI	APA-induced acute liver injury
APA	Acetaminophen
AST	Aspartate aminotransferase
ATP	Adenosine triphosphate
CQ	Chloroquine diphosphate salt
ER	Endoplasmic reticulum
GCM	Glycycoumarin
GSH	Glutathione
H&E	Hematoxylin and eosin
NAPQI	*N*-acetyl-*p*-benzoquinone imine
NAC	*N*-acetyl-L-cysteine
PCNA	Proliferating cell nuclear antigen
SEM	Standard errors of the mean
SULT	Sulfotransferase
TEM	Transmission electron microscope
UGT	UDP-glucuronosyltransferase
UPR	Unfolded protein response
